# Two‐Step Palatal Shield Technique Using Flowable Composite Resin for Donor‐Site Protection Following De‐Epithelialized Free Gingival Grafting: A Case Report Illustrating a Novel Technique

**DOI:** 10.1155/crid/3572064

**Published:** 2026-08-13

**Authors:** Lucas Raineri Capeletti, Marcelo Pereira Nunes, Washington Macedo Santana, João Antônio Chaves de Souza, Paulo Vitor Fernandes Braz, France Lambert, Carlos Alexandre Soares Andrade

**Affiliations:** ^1^ Department of Periodontology, Universidade Federal de Goiás (UFG), Goiânia, Goiás, Brazil, ufg.br; ^2^ Proimperio Institute, São Paulo, Brazil; ^3^ INFace, Private Practice, Goiânia, Goiás, Brazil; ^4^ Department of Dentistry, Universidade de Brasília (UnB), Brasília, Distrito Federal, Brazil, unb.br; ^5^ Department of Periodontology, Oro-Dental and Implant Surgery Faculty of Medicine, CHU of Liège, University of Liège, Liège, Belgium, ulg.ac.be

**Keywords:** case report, connective tissue, dental implants, palate, periodontics, tissue grafting

## Abstract

Although multiple protective techniques have been proposed for the palatal donor‐site management after de‐epithelialized free gingival graft (DFGG) procedures, many increase patient discomfort, require additional resources, or prolong surgical time. This case report introduces a novel two‐step palatal shield (TSPS) technique using flowable composite resin, designed to simplify donor‐site protection, improve patient comfort, and enhance postoperative healing. A female patient, 43 years old, underwent DFGG harvesting from the palate for immediate implant placement with esthetic tissue augmentation. A customized palatal shield was fabricated chairside using flowable composite resin to replicate the presurgical anatomy of the donor site. The shield was tooth‐supported and suture‐free, allowing rapid insertion and stable wound coverage. The patient was followed postoperatively, with evaluations at suture removal and extended follow‐up up to 9 months. The patient reported minimal pain or discomfort at the donor site, and clinical examination showed favorable epithelialization and soft tissue healing. The TSPS technique represents a novel, practical approach for donor‐site management utilizing affordable materials. Its immediate preoperative fabrication, tooth‐supported stabilization, and suture‐free design may simplify the clinical workflow, help reduce operative time, and potentially enhance patient comfort, whereas precise anatomical adaptation appears to promote favorable clot stabilization and healing.

## 1. Background

Mucogingival surgery is a key component in both implant dentistry and periodontal procedures, serving not only cosmetic purposes but also critical functional roles in implant therapy [1–3]. Soft tissues surrounding dental implants are essential for maintaining long‐term stability, success, health, and esthetic outcomes [4, 5]. Inadequate gingiva and peri‐implant mucosa can compromise both implant function and peri‐implant tissue integrity [6, 7]. Procedures like de‐epithelialized free gingival graft (DFGG) and subepithelial connective tissue graft (CTG) are employed to augment the width and thickness of keratinized tissue, typically using autologous tissue harvested mainly from the palate or maxillary tuberosity, though alternative donor sites can also be considered [8, 9]. Increasing keratinized tissue has been linked to reduced plaque accumulation, lower peri‐implant inflammation, and preservation of crestal bone levels, all of which enhance implant survival and long‐term success [10–12]. In particular, DFGGs are widely evidenced as the most effective technique for expanding peri‐implant keratinized mucosa and restoring gingival architecture, whereas CTGs can be applied to treat gingival recessions and optimize soft tissue contours [13–15].

The healing process and patient morbidity following DFGG harvesting are often viewed as complex to manage by clinicians and traumatic for patients [16]. The primary source of postoperative discomfort arises from the palatal donor site, where tissue harvesting leaves an open wound that heals by secondary intention over approximately 2–4 weeks [16, 17]. Healing at the donor site involves complex biological processes, including fibroblast proliferation, collagen synthesis, angiogenesis, epithelial cell regeneration, and revascularization, all of which contribute to gradual tissue closure and restoration of mucosal integrity [18, 19]. Despite these healing mechanisms, patients frequently experience the highest level of pain on the first postoperative day, which typically decreases to presurgical levels within 2 weeks [20]. This discomfort is exacerbated by mechanical irritation from the tongue and food during the healing period, as well as the necessity of maintaining two surgical sites (donor and recipient), which inherently increases morbidity [21, 22]. Additional complications associated with DFGG harvesting include postoperative hemorrhage, bone exposure, and high levels of pain [23–25]. Although alternatives to autologous grafts exist, none fully replicate their biological and structural properties, making autologous grafting the gold standard despite the temporary pain and functional limitations it imposes [9, 26]. Importantly, the patient′s perception of postoperative discomfort can influence their willingness to undergo future soft tissue procedures, highlighting the need for careful surgical planning, effective pain management, and clear patient education regarding the expected healing trajectory and potential complications [27–29].

To minimize postoperative morbidity and accelerate healing at the palatal donor site following DFGG procedures, a variety of protective techniques have been developed [28]. A commonly used approach involves placing an absorbable gelatin sponge or collagen plug over the wound and securing it with sutures, effectively reducing bleeding, pain, and exposure to mechanical irritation [30]. Alternative strategies include the application of leukocyte‐ and platelet‐rich fibrin (L‐PRF) [31], which has been shown to enhance epithelialization and decrease postoperative discomfort, as well as cyanoacrylate adhesives [32, 33], hyaluronic acid [32], or combinations of those with gelatin sponges to further improve wound protection and patient comfort [34]. Physical barriers such as acrylic or resin stents, collagen membranes, and propylene meshes also serve to shield the donor site, although some may increase cost or present challenges in stability [27, 35, 36]. Adjunctive measures, including photobiomodulation, topical anti‐inflammatory sprays, and biostimulants, have been explored to support healing, though their effects on pain reduction vary [37–40]. Despite this range of interventions, some clinicians still rely on natural uncovered healing, highlighting ongoing uncertainties about the optimal method for every patient [39]. Overall, protecting the donor area is a critical component of DFGG procedures, with evidence suggesting that combining mechanical barriers with biologically active materials can significantly reduce postoperative pain, limit complications, and promote faster epithelial recovery [28].

Although numerous techniques have been proposed to protect the palatal donor site, each presents important limitations. Many require suturing for stability, which increases procedure time and may cause postoperative discomfort [41, 42]. Adjuncts such as L‐PRF [31], laser [43], ozone [44–46], amelogenins [47], and adhesives [48] demand specialized equipment, training, or incur additional costs, whereas laboratory‐fabricated barriers and stents necessitate multiple appointments and still provide limited stabilization. Moreover, the effectiveness of options such as L‐PRF [49], suturing [30], cyanoacrylate [33], or stents [22] remains inconsistent, and no method has demonstrated universal superiority. Recent studies have investigated the use of flowable composite resin as a protective shield over collagen sponges or directly on the suturing of the donor area to mitigate postoperative pain after DFGG harvesting [42, 50, 51]. These approaches have shown advantages in patient comfort, ease of application, cost‐effectiveness, and facilitation of donor‐site healing [41, 42]. However, they also present limitations, including the need for suturing to secure the resin shield, poor adaptation to interdental spaces, limited accommodation for blood or hemostatic materials, and reduced anatomical conformity due to postsurgical fabrication [52]. Additionally, palatal protectors and sutures can be too thick or cause discomfort in the tongue [53], whereas cyanoacrylate, full‐palate plates, and palatal knots may further contribute to patient trauma [35].

This case report is aimed at introducing the novel two‐step palatal shield (TSPS) technique with flowable resin that requires no suturing, with immediate presurgical fabrication of the palatal protector followed by postsurgical placement, leaving space for hemostatic materials, replicating the presurgical anatomy of the palate and enhancing contact with interdental areas, with the goal of improving postoperative comfort and promoting more effective donor site healing.

## 2. Case Presentation

### 2.1. Patient Selection

This study adhered to the SCARE guidelines [54]. A single patient seeking implant‐supported treatment and requiring DFGG from the palate was considered for this case report. The case was conducted in a private dental clinic in Brasília (Federal District), Brazil, and patient selection took place in 2024. The patient agreed to have her case published, and written informed consent was obtained prior to treatment. The included patient was 43 years old, nonsmoker, systemically healthy, in need of an implant in the anterior esthetic zone, and with a palate suitable for placement of the protective shield. Exclusion criteria included pregnancy or breastfeeding, history of periodontitis, history of chemotherapy or radiotherapy in the head or neck region, use of intravenous bisphosphonates, presence of oral lesions on the palate, use of dental prosthesis covering the palate, or any other palatal condition contraindicating placement of the shield.

The patient presented to the authors′ private clinic. She underwent a comprehensive preoperative examination, which included detailed clinical evaluation, intraoral and extraoral photography, cone‐beam computed tomography (CBCT), and intraoral scanning to accurately assess the donor and recipient sites. Based on these assessments, a personalized treatment plan was developed and alternative therapeutic options were thoroughly discussed, including their potential risks, benefits, and expected outcomes. After careful consideration, the patient elected to proceed with the proposed treatment approach. Prior to surgery, the patient received thorough periodontal therapy to ensure favorable oral health, and individualized oral hygiene instructions were provided to support healing and reduce the risk of postoperative complications. This careful preparatory phase aimed to optimize both surgical outcomes and long‐term implant success. The surgery occurred in January 2025. Surgical and prosthetic procedures were performed by a single experienced clinician (L.R.C.). The case was fully documented with clinical photography and followed for 9 months. Information about the patient is summarized in Table 1.

**Table 1:** Case description, surgery details, and follow‐up time.

Age (years)	Sex	Type of surgery	Donor side	Gingival biotype	Smile line	Overall follow‐up (months)
43	Female	Immediate implant placement in the esthetic zone	Palate	Thin	Medium	9

### 2.2. Palatal Shield Fabrication

**Figure 1:**
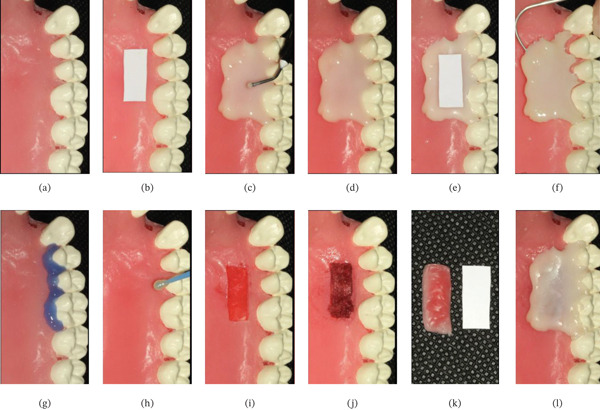
Sequential demonstration of the two‐step palatal shield technique using flowable composite resin on a dental mannequin model. (a) Preoperative initial view of the palatal area. (b) Positioning of the paper template to measure and outline the planned graft harvesting area. (c) Chairside application of flowable composite resin over the selected palatal donor site and into the interdental spaces of adjacent teeth to ensure optimal retention and stability. (d) Light‐curing of the flowable composite resin to finalize the prefabricated shield. (e) Evaluation of the prefabricated shield in place, ensuring boundaries are maintained at least 2 mm from the planned surgical boundaries. (f) Removal of the customized palatal shield using a dental probe prior to surgical disinfection. (g) Enamel acid etching of the interdental and palatal surfaces of adjacent teeth with 37% phosphoric acid. (h) Application of a universal adhesive system in a uniform layer. (i) View of the palatal donor site immediately following simulated graft harvesting. (j) Placement of a hemostatic sponge within the open wound. (k) Side‐by‐side visual comparison between the paper template and the harvested graft. (l) Final placement of the customized palatal shield.

**Figure 2:**
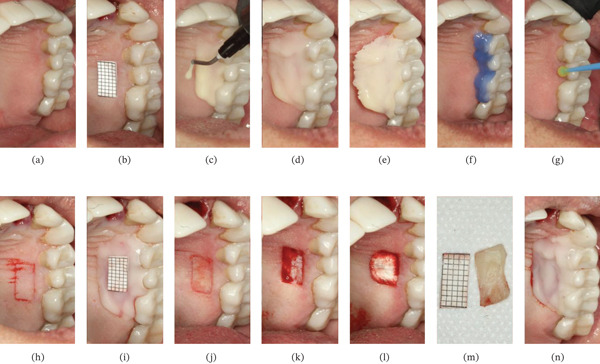
Clinical application of the two‐step palatal shield technique in the patient undergoing de‐epithelialized free gingival graft harvesting. (a) Preoperative clinical view of the palatal donor site. (b) Positioning of a grid paper template to measure and delineate the planned donor area. (c) Application of flowable composite resin over the palatal region and interproximal spaces of adjacent teeth to replicate the natural presurgical anatomy. (d) Light‐curing of the flowable composite resin. (e) Careful removal of the customized palatal shield with a dental probe for immersion in a 2% chlorhexidine solution. (f) Enamel acid etching of the interproximal and palatal surfaces of Teeth 14–16 using 37% phosphoric acid. (g) Application of a universal adhesive system in a uniform layer. (h) Initial incision and outline of the palatal donor site. (i) Placement of the prefabricated shield to verify a proper 2‐mm safety margin around the planned surgical boundaries. (j) Refinement of the palatal incision according to the grid paper template. (k) Clinical view of the palatal donor site immediately after the graft is harvested. (l) Placement of a hemostatic collagen sponge into the open wound. (m) Visual comparison between the grid paper template and the actual harvested graft. (n) Final palatal shield fixed in place and stabilized.

The first step in the TSPS technique involves presurgical fabrication of the shield, which was performed immediately before the surgical procedure (Figures 1 and 2). The donor area was carefully selected, and a flowable composite resin (Opallis Flow 72% FGM, Joinville, State of Santa Catarina, Brazil) was applied over the palatal region intended for harvesting. The interdental spaces of adjacent teeth were included to improve retention and stability. This application allowed precise replication of the natural palatal anatomy, ensuring satisfactory adaptation and minimizing pressure on surrounding tissues. The shield boundaries were maintained 2 mm from the planned surgical site to allow proper sealing, protection, and stability. After light‐curing (VALO Cordless, Ultradent Products Inc., South Jordan, Utah, United States) for 60 s, according to the manufacturer′s instructions, the shield was removed with a probe. This prefabrication ensures the final shield conforms closely to the patient′s anatomy, providing a customized protective barrier for the donor site. Subsequently, the palatal shield was immersed in a 2% chlorhexidine solution (Colgate‐Palmolive, New York, New York, United States) for at least 15 min.

The second step involves postsurgical placement of the palatal shield. To obtain the necessary connective tissue, a DFGG was harvested and extraorally de‐epithelialized, making this technique particularly indicated for cases involving DFGG harvesting with an open palatal wound. After graft harvesting, stabilization of the graft and suturing of the recipient site were performed to allow for natural clot stabilization at the donor area, which typically occurs after approximately 10 min. No suction was applied to the donor site to avoid disruption of the clot. This interval facilitated drying and the enamel surface in the interproximal and palatal regions were etched with 37% phosphoric acid for 30 s, followed by thorough rinsing for an additional 30 s. Subsequently, a universal adhesive (Single Bond Universal, 3M ESPE, St. Paul, Minnesota, United States) was applied in a uniform layer and light‐cured for 20 s. Flowable composite was applied to the teeth, the palatal shield was inserted, and the whole complex was light‐cured for 60 s. The stabilization of the shield without the need for suturing reduces trauma and postoperative discomfort. Because the shield replicates the presurgical palatal anatomy, it leaves sufficient space for the healing tissue to expand and regain its original thickness while maintaining protection and stability during the early stages of healing.

**Figure 3:**
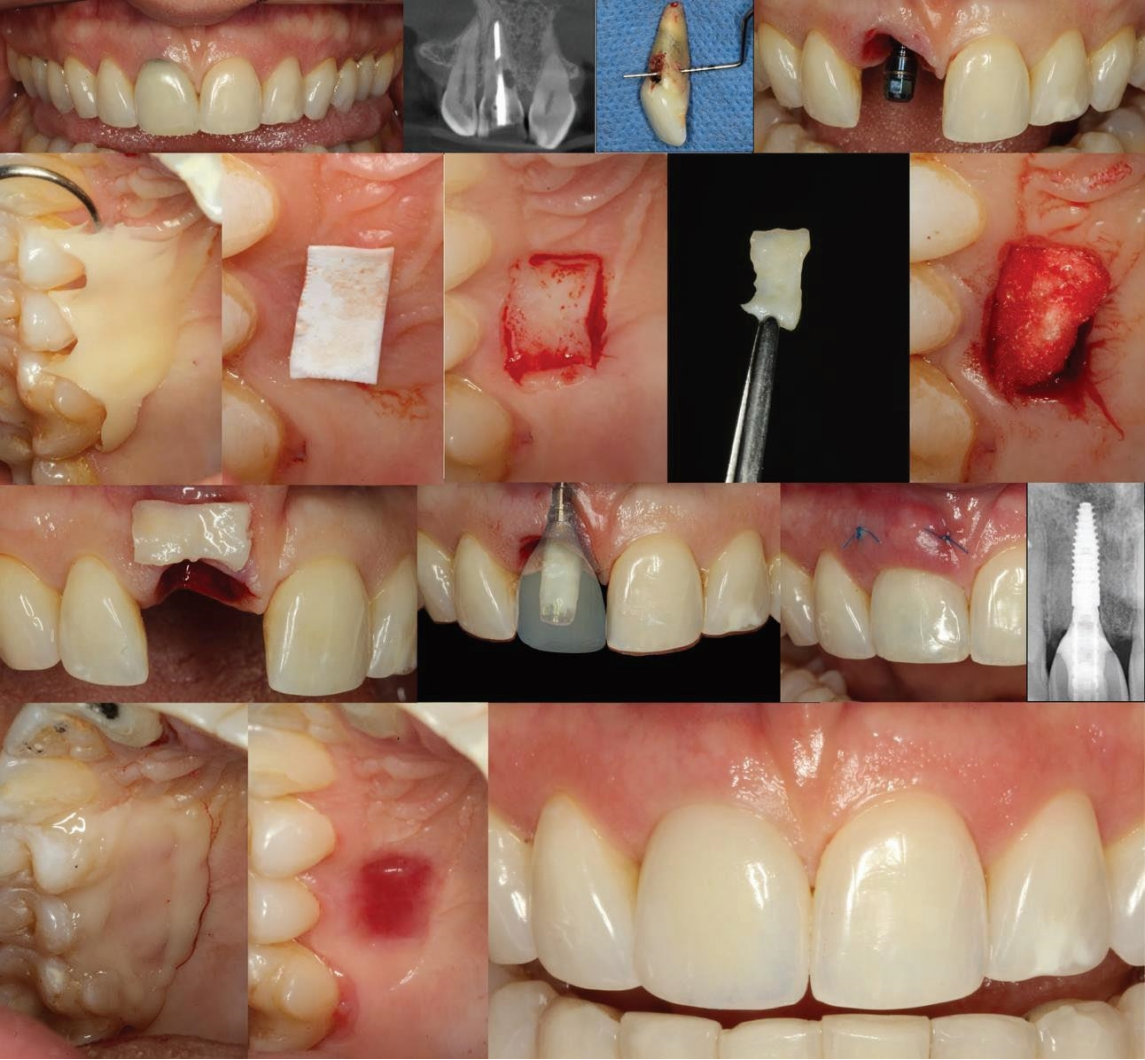
The use of the two‐step palatal shield technique for palate protection in a treatment of a root resorption in an anterior tooth. Clinical photographs, CBCT, and radiograph of the patient showing its management with immediate implant placement, de‐epithelialized free gingival grafting, and final crown.

A 43‐year‐old female patient presented to the clinic after being referred for management of root external resorption affecting Tooth 11 (Figure 3). She reported that the cervical area of the tooth had been gradually darkening and that she was experiencing pain in the region. A CBCT scan confirmed the presence of significant root resorption extending from the mesial toward the buccal sites, compromising the long‐term prognosis of the tooth. The patient had undergone 10 years of clinical and tomographic follow‐up, during which endodontic treatments and interventions were performed to prolong tooth survival. Despite these efforts, the resorption process continued. Although the tooth remained clinically favorable, the progressive resorption posed an increasing risk of fracture and infection, leading to the decision to proceed with surgical treatment. Given the esthetic importance of the anterior maxillary region and the extent of the resorptive lesion, the interdisciplinary treatment plan involved minimally invasive extraction of Tooth 11, immediate implant placement, soft tissue augmentation using a DFGG, and immediate provisionalization using the patient′s own natural crown to preserve esthetics. Surgery was initiated with an atraumatic approach, employing a flapless intrasulcular incision and the use of periotomes to carefully detach periodontal ligament fibers, thereby minimizing trauma to the surrounding hard and soft tissues. Upon extraction, the tooth was found to exhibit extensive resorption involving both mesial and buccal surfaces. An implant (BLT NC SLActive, 3.3 × 12 mm; Straumann, Basel, Switzerland) was placed into the socket with primary stability. A temporary crown was fabricated by capturing the buccal shell of the patient′s extracted crown onto a NC immediate temporary abutment (Narrow CrossFit, Straumann, Basel, Switzerland) with flowable resin, and composite resin was subsequently added to refine the emergence profile and ensure a harmonious gingival contour.

To enhance peri‐implant soft tissue volume and promote a natural gingival appearance, a DFGG was harvested from the palatal region adjacent to the maxillary second premolar. A 1‐mm thin layer of tissue was carefully excised from the palate and de‐epithelialized extraorally to expose the underlying connective tissue, which was then adapted to the recipient site at Tooth 11 and secured with sutures for stability. The extraction socket was augmented with deproteinized porcine bone mineral (DPBM) (THE Graft, Purgo Biologics, Seongnam, Korea) to support bone regeneration and enhance implant integration. Meanwhile, the donor site was managed by placing a collagen sponge (Hemospon Hemostatic Sponge, Maquira, Brazil) and protecting the region with the custom palatal shield, fabricated chairside with flowable resin over Teeth 14–16, thereby potentially improving postoperative comfort and healing. Postoperative recovery was uneventful, with the patient returning after 14 days for suture removal, at which time the palatal donor site demonstrated favorable healing. A follow‐up at 9 months confirmed that both the palate and peri‐implant tissues were healthy and well‐contoured, and the patient verbally reported no discomfort during the healing period, although objective pain scales were not recorded. The final prosthetic crown was then delivered on Tooth 11, restoring esthetics, function, and patient confidence while achieving a stable and natural integration with the surrounding dentition.

## 3. Discussion and Conclusion

This case report illustrates the novel TSPS technique, representing a highly practical approach to protect the palatal donor site following DFGG harvesting. To the best of our knowledge, no previous study has described this exact method of utilizing flowable composite resin to fabricate a customized palatal shield directly in the clinical setting. The TSPS is fabricated chairside immediately prior to surgery, which avoids contamination with blood and saliva, shortens operative time, and enhances clinical efficiency without requiring laboratory assistance, additional appointments, or special training. Relying solely on inexpensive, readily available materials, this simple technique accurately reproduces the exact presurgical palatal anatomy. This enables precise blood clot stabilization and guided healing without needing sutures or material placement within the wound, ensuring accurate adaptation and hemostasis. The technique demonstrates significant versatility across various clinical indications, such as peri‐implant soft tissue augmentation or sequential grafting phases, with minor variations introduced based on the extent of harvesting. Furthermore, the TSPS provides stable protection even in smaller grafts and allows straightforward postoperative adjustments. Across different scenarios, including the present case, palatal healing was uneventful, with the patient demonstrating favorable epithelialization and reporting minimal pain. Overall, this highly accessible option can be easily adopted in everyday clinical practice to minimize postoperative discomfort and reduce surgical time.

Compared with the TSPS technique, alternative palatal protection methods present many clinical limitations. L‐PRF techniques increase procedural complexity and patient discomfort by requiring blood collection, specialized training, and centrifuges [31, 49, 55]. Similarly, laser or ozone applications limit routine clinical applicability due to their demand for extra visits, specialized devices, and training [43–45]. Furthermore, laboratory‐fabricated full‐palate devices often compromise patient comfort, interfere with oral functions, and increase costs and chair time [12]. Unlike the TSPS, zinc‐containing surgical stents require specialized equipment, can be overly thick, and compromise patient comfort [53]. Similarly, traditional suturing increases surgical time, can cause pressure‐related pain, and may loosen, threatening wound protection [30]. In contrast, the TSPS technique is preoperatively fabricated in minutes, tooth‐supported, suture‐free, and minimally invasive. Relying only on standard clinical materials, it simplifies the clinical workflow and improves patient comfort.

Several studies have evaluated resin‐based palatal shields for donor‐site protection, though they present structural differences compared with the TSPS technique. One randomized clinical trial utilized a postoperative flowable composite shield secured with sutures and a collagen sponge [50], reporting reduced pain and analgesic use compared with other techniques using conventional suturing alone [31, 43, 44, 55, 56]. However, unlike the TSPS, such studies report the fabrication occurring postoperatively within an active, bleeding surgical field. Similarly, investigations comparing flowable shields to periodontal dressings like Coe‐Pak reported improved pain control, but these protocols required suturing and lacked tooth support, which prolonged chair time [42, 58]. Lastly, a trial evaluating multiple modalities noted accelerated healing with flowable resin, yet the shield still relied on sutures and adjunctive sponges rather than a self‐stabilizing, suture‐free design [41]. Similarly, composite shields reinforced with Alvogyl required postoperative fabrication and auxiliary materials, reducing practical efficiency [51]. Although a recent protocol utilized a suture‐free, tooth‐supported shield, it was fabricated postoperatively, serving only as a secondary element of the procedure rather than the primary focus of the study [52]. In contrast, the present two‐step technique overcomes these limitations by being fabricated preoperatively, directly supported by teeth, and requiring no sutures or additional materials, thereby simplifying the clinical workflow while ensuring accurate anatomical adaptation and effective donor‐site protection.

The present case report has several key strengths. Foremost, it introduces a completely novel approach for palatal donor‐site protection that has not been previously described, highlighting its originality in clinical practice. The TSPS is practical and easy to implement, as it can be fabricated and positioned chairside within minutes using widely available materials, without requiring specialized equipment or extensive training. Its suture‐free, tooth‐supported design appears to increase stability, may reduce operative time, and is designed to potentially minimize patient discomfort, whereas the precise anatomical adaptation helps stabilize the blood clot and may enhance wound healing. The technique relies on inexpensive materials and avoids laboratory fabrication or outsourcing. Additionally, the patient was followed for up to 9 months, providing longer term insight into palatal healing than is commonly reported in donor‐site studies. In summary, this two‐step approach integrates anatomical precision with mechanical support, aimed at optimizing patient comfort and donor site protection while enhancing clinical efficiency and reducing costs for both the patient and the clinician. Despite these advantages, this case report has important limitations. The report is limited to this case, which restricts the generalizability of the findings and prevents statistical analysis of outcomes such as pain, discomfort, or wound healing rate. The absence of comparisons with other donor‐site protection techniques cannot quantify relative effectiveness. A limitation of the TSPS is the difficulty of cleaning it while in place, which may increase inflammation in some cases, making postoperative mouth‐rinse use essential. This risk is limited by the short duration of use and the close adaptation of the shield to the palate. Furthermore, as highlighted by its design, a critical limitation is the dependency of the TSPS technique on the presence of adjacent teeth for stabilization. In cases with minimal or no teeth around the donor site, the applicability of this technique is highly restricted. In such situations, clinicians can try alternative methods with flowable composite, such as creating a shield stabilized with sutures or placed directly on the suturing of the donor area. In addition, although healing was favorable, and the patient reported minimal discomfort, a notable limitation of this report is the lack of a standardized method of pain assessment, such as a visual analog scale (VAS) or validated patient‐reported outcome measures (PROMs). Without these standardized metrics, the claims regarding pain reduction rely on subjective clinical observations. Furthermore, potential variability in surgical skill or patient factors could influence the results. Therefore, larger, controlled studies incorporating standardized pain metrics (e.g., VAS scores) and extended monitoring are needed to objectively validate the reproducibility, clinical effectiveness, and patient experience associated with this novel technique.

## Author Contributions

L.R.C. contributed to conceptualization, methodology, and investigation. M.P.N., W.M.S., and J.A.C.S. were responsible for methodology and validation. P.V.F.B., F.L., and C.A.S.A. contributed to methodology, visualization, and writing the original draft. All authors were involved in writing, reviewing, and editing the final manuscript.

## Funding

No funding was received for this manuscript.

## Disclosure

All authors have read and approved the final version of the manuscript. L.R.C. had full access to all of the data in this study and takes complete responsibility for the integrity of the data and the accuracy of the data analysis.

## Consent

As a single anonymized case report, this study was exempt from formal institutional ethical approval. However, written informed consent was obtained from the patient for both the treatment and the publication of this case, including all accompanying figures.

## Conflicts of Interest

The authors declare no conflicts of interest.

## Supporting information


**Supporting Information** Additional supporting information can be found online in the Supporting Information section. 

## Data Availability

The data that support the findings of this study are available on request from the corresponding author. The data are not publicly available due to privacy or ethical restrictions.
