# Supplementary material for: Two‐Step Palatal Shield Technique Using Flowable Composite Resin for Donor‐Site Protection Following De‐Epithelialized Free Gingival Grafting: A Case Report Illustrating a Novel Technique
Source: Case Rep Dent. 2026 Aug 13;2026:3572064. doi: 10.1155/crid/3572064 (PMC13471641; doi:10.1155/crid/3572064)
Supplement: Supplementary file 1 [file CRID-2026-3572064-s001.pdf]

CARE Checklist Mapping Table

CARE Item # & Topic	CARE Guideline Description	Manuscript Location & Supporting Details	Line Range
1. Title	The diagnosis or intervention of primary focus followed by the words 'case report'.	Included in full title: 'Two-step palatal shield technique... a case report illustrating a novel technique'.	Lines 1-3
2. Key Words	2 to 5 key words that identify diagnoses or interventions, including 'case report'.	Keywords listed: 'Palate, periodontics, dental implants, connective tissue, tissue grafting, case report.'	Line 72
3a. Abstract Intro	What is unique about this case and what does it add to the literature?	Focuses on the clinical challenge of open palatal donor wounds and introduces the TSPS technique using flowable resin.	Lines 54-58
3b. Abstract Findings	Main symptoms and/or important clinical findings.	Notes 43-year-old female patient needing immediate implant and esthetic soft-tissue augmentation (DFGG).	Lines 58-60
3c. Abstract Intervention	Main diagnoses, therapeutic interventions, and clinical outcomes.	Pre-fabricated chairside palatal shield, suture-free and tooth-supported. Favorable healing and minimal discomfort over 9 months.	Lines 60-66
3d. Abstract Conclusion	Main 'take-away' lesson(s) from the case.	TSPS is a practical, suture-free, and affordable technique that reduces operative time and enhances patient comfort.	Lines 66-71
4. Introduction	One or two paragraphs summarizing background and why this case is unique.	Background on mucogingival surgery, morbidity of secondary intention healing, and limitations of existing protective techniques.	Lines 73-153
5a. Patient Info	De-identified patient specific information.	A 43-year-old female, non-smoker, systemically healthy.	Lines 160-161
5b. Primary Concerns	Primary concerns and symptoms of the patient.	Gradual darkening of the cervical area of tooth 11 accompanied by localized pain.	Lines 251-252
5c. History	Medical, family, and psycho-social history including relevant genetic information.	Systemically healthy, non-smoker. No tobacco, relevant history is localized root resorption under 10 years of monitoring.	Lines 160, 254-255
5d. Past Interventions	Relevant past interventions with outcomes.	10 years of clinical and tomographic follow-up with endodontic treatments to prolong survival of tooth 11.	Lines 255-256
6. Clinical Findings	Significant physical examination (PE) and important clinical findings.	Confirmed thin gingival biotype, medium smile line, and extensive root resorption compromising the long-term prognosis.	Lines 250-254<br>Table 1
7. Timeline	Historical and current information from this episode of care organized as a timeline.	Surgical event (Jan 2025), post-op suture removal (14 days), and long-term restorative outcome (9 months).	Lines 250-291

8a. Diagnostic Testing	Diagnostic testing (PE, laboratory testing, imaging, surveys).	Comprehensive examination: clinical evaluation, intraoral/extraoral photos, CBCT scans, and intraoral scans.	Lines 168-171, 252-253
8b. Diagnostic Challenges	Diagnostic challenges (access, financial, cultural).	No diagnostic challenges reported; full testing was successfully accessible in author's private clinics.	N/A
8c. Diagnosis	Diagnosis (including other diagnoses considered).	Severe external root resorption affecting tooth 11.	Lines 250-251
8d. Prognosis	Prognosis (where applicable).	Compromised long-term prognosis of tooth 11, indicating immediate extraction and implant therapy.	Lines 253-254
9a. Intervention Type	Types of therapeutic intervention (pharmacologic, surgical, preventive, self-care).	Surgical: Tooth 11 extraction, immediate implant, socket bone graft (Purgo DPBM), DFGG soft tissue graft, and chairside TSPS.	Lines 260-284
9b. Intervention Admin	Administration of therapeutic intervention (dosage, strength, duration, technical steps).	Detailed chairside protocol for TSPS: Opallis Flow 72% resin, VALO curing (60s), 2% chlorhexidine, 37% phosphoric acid etching.	Lines 185-216
9c. Intervention Changes	Changes in therapeutic intervention (with rationale).	No changes required. The planned surgical and protective protocol was successfully executed as designed.	N/A
10a. Outcomes	Clinician and patient-assessed outcomes.	Uneventful healing, healthy well-contoured palate/peri-implant tissue at 9 months. Patient verbally reported no discomfort.	Lines 284-291
10b. Follow-up results	Important follow-up diagnostic and other test results.	Favorable epithelialization and soft-tissue contour shown via clinical follow-up at 14 days and 9 months.	Lines 284-288
10c. Adherence	Intervention adherence and tolerability (how was this assessed?).	Assessed clinically during suture removal (14 days) and 9-month recall; no clinical complications or device loss occurred.	Lines 285-288
10d. Adverse Events	Adverse and unanticipated events.	Recovery was completely uneventful with no adverse reactions, device failure, or bleeding complications.	Lines 284-285
11a. Strengths/Limits	Scientific discussion of strengths AND limitations associated with the case.	Strengths: Originality, 9-month follow-up. Limitations: N=1, lack of comparisons, no objective VAS score, tooth-dependency.	Lines 349-382
11b. Lit Review	Discussion of relevant literature with references.	Exhaustive comparison with traditional protective techniques (sutures, stents, dressings, L-PRF, laser/ozone).	Lines 317-348
11c. Scientific Rationale	Scientific rationale for conclusions (including assessment of possible causes).	Discussion on why preoperative, suture-free design promotes clot stability, protects wound, and improves comfort.	Lines 301-308, 350-356

11d. Take-away Lessons	Primary take-away lessons in a single-paragraph conclusion.	TSPS integrates anatomical precision and mechanical support to optimize clinical workflows and patient outcomes.	Lines 359-362
12. Patient Perspective	The patient shares their perspective in 1-2 paragraphs on treatments received.	The patient verbally reported high satisfaction and no discomfort. No separate personal essay/statement is included.	Line 288
13. Informed Consent	Did the patient give informed consent? Please provide if requested.	Explicitly declared that written informed consent was obtained from the patient for treatment and case publication.	Lines 49-51, 159-160
